# Questions Given by Maryland State Board of Dental Examiners, May, 1898

**Published:** 1898-07

**Authors:** 


### Editorial, Etc.

### QUESTIONS GIVEN BY MARYLAND STATE BOARD OF DENTAL EXAMINERS, MAY, 1898.

### PATHOLOGY, THERAPEUTICS AND MATERIA MEDICA.

1. What is Pathology?
2. What is Materia Medica?
3. What is Therapeutics?
4. What is Pulpitis?
5. Describe its cause, symptoms and treatment.
6. Describe what you know of Pyorrhœa Alveolaris, its etiology and treatment.
7. What is Stomatitis?
8. What remedies are used in its treatment?
9. What is an Ulcer?
10. What is an Apthous Ulcer?
11. What is its cause and treatment?
12. Why does the introduction of Mercury into the system cause the saliva to flow more profusely?
13. What is the technical name for Mercury?
14. Write a prescription containing three or more ingredients?
15. What is an Escharotic?
16. What is the meaning of Ptyalism?
17. Describe Inflammation.
18. What is dental Caries? Its cause?
19. What is a Styptic? Name several.
20. What is an Antiphlogistic?
21. What is the meaning of Prophylactic?
22. What is Carbolic Acid, its origin and uses?
23. What is Muriatic Acid?

### OPERATIVE DENTISTRY.

1. Give your method of preserving the incisors when afflicted by caries at the age of twelve years; name the materials you would use.
2. Give your method of treating when the nerve is nearly exposed?
3. Name Drug or Drugs used in destroying nerves of teeth and how applied.
4. What is the diagnostic sign of hyper-sensitive dentine?
5. What is the cause of Periostitis?
6. What is the treatment of Periostitis?
7. Give your method of treating diseased and abscessed roots of teeth; and your mode and material for filling same?
8. When the gum has very much receded on the labial and not on the lingual surface, how do you protect a cavity that extends under the gum on the labial surface and what gold would you use?
9. Describe the action of arsenious acid on the Dental Pulp?
10. In respect to properties of cohesive and non-cohesive Gold, what is the distinction between them?
11. What materials are employed for filling root canals?
12. How is amalgam introduced and condensed into a cavity?
13. How are the different plastic materials now in use introduced into the cavities; name four of the materials?
14. How can hemorrhage from tooth extraction be arrested?
15. What is the treatment of irritability of the Dental Pulp?
16. What is temperament—of what use is a knowledge of it to a Dentist?
17. You will be expected to operate, by inserting a gold filling.

### CHEMISTRY.

1. What is the object of the science of Chemistry?
2. What is matter?
3. What is force?
4. Into how many conditions is matter divided; and what are they?
5. Describe the characteristic properties of matter in solid, liquid and gaseous states.
6. How many elements are there in nature?
7. Name some of the most important, some of lesser importance, and some of least importance.
8. Why are they not of equal importance?
9. What elements at ordinary temperature are solid; what are liquid and what are gases?

10. What element is present on the earth in the greatest quantity?
11. What is atmospheric air?
12. How is carbon found in nature?
13. State the physical and chemical properties of carbon in its three allotropic modifications.
14. What is flame? Explain the structure of an ordinary flame.

ANATOMY.

1. Name the bones of the face.
2. What are the principal muscles of mastication?
3. Describe the fifth nerve, and give its branches.
4. Of what parts are the teeth composed?
5. Give the names of the permanent teeth, and time of eruption.
6. Name the organs of insalivation.
7. What cavity does the superior maxillary contain?
8. Where is the sphenoidal fissure, and what vessels pass through it?
9. What is the structure of enamel? Of dentine?
10. Describe the dental pulp.

PHYSIOLOGY.

1. What are the primary stages of digestion in which the mouth is concerned?
2. Describe the circulation of the blood, beginning at the right ventricle.
3. What proportion of the body's weight does the blood compose?
4. What is the temperature of the blood?
5. What change takes place in the blood as it passes through the lungs?
6. What are the functions of the peridental membrane?
7. Describe the process of digestion.
8. Define reflex action; give example.

MECHANICAL DENTISTRY AND METALLURGY.

1. What is Dental Prosthesis? With what does it deal?
2. When teeth are lost, what anatomical changes occur?
3. Give the requisites of an ideal impression material.
4. What objections to such as are in ordinary use?
5. What is the difference between the plaster used by the dentist and the ground plaster used as a fertilizer?
6. What shapes should be given the body of the model for sand moulding?

7. What is moulding sand? How tempered and used?
8. What qualities are important in a plate for dental purposes?
9. What metals are used in such work?
10. What metals are used in a pure state? What metals alloyed?
11. How could you cut cavities in porcelain teeth for the insertion of gold fillings?
12. What is meant by metallo-plastic work?
13. What is meant by stanno-plastic work?

ORAL SURGERY.

1. What disease is the Antrum of Highmore subject to? Give method of treatment when all the teeth are sound and it is not advisable to extract any.
2. How would you diagnose dislocation of the lower jaw? How would you treat it?
3. How would you diagnose fracture of the maxilla? State treatment.
4. How would you make an inter-dental splint?
5. Describe an alveolar abscess. Give method of treatment.

---

